# The Role of Circulating Tumor Cells in the Metastatic Cascade: Biology, Technical Challenges, and Clinical Relevance

**DOI:** 10.3390/cancers12040867

**Published:** 2020-04-03

**Authors:** Hassan Dianat-Moghadam, Mehdi Azizi, Zahra Eslami-S, Luis Enrique Cortés-Hernández, Maryam Heidarifard, Mohammad Nouri, Catherine Alix-Panabières

**Affiliations:** 1 Stem Cell Research Center, Tabriz University of Medical Sciences, Tabriz 51368, Iran; Dianat.hassan@gmail.com (H.D.-M.); Nourimd@yahoo.com (M.N.); 2 Student Research Committee, Tabriz University of Medical Sciences, Tabriz 51368, Iran; 3 Proteomics Research Center, Tabriz University of Medical Sciences, Tabriz 51368, Iran; Mehdi.azizi6815@gmail.com; 4 Laboratory of Rare Human Circulating Cells (LCCRH), University Medical Centre of Montpellier, UPRES, EA2415, 34093 Montpellier, Francele-cortes-hernandez@chu-montpellier.fr (L.E.C.-H.); 5 Drug Applied Research Center, Tabriz University of Medical Sciences, 51368 Tabriz, Iran; maryam.heydarifard@gmail.com

**Keywords:** circulating tumor cells, metastasis, cancer stem cells, dormancy, immune escape, therapy resistance, liquid biopsy

## Abstract

Metastases and cancer recurrence are the main causes of cancer death. Circulating Tumor Cells (CTCs) and disseminated tumor cells are the drivers of cancer cell dissemination. The assessment of CTCs’ clinical role in early metastasis prediction, diagnosis, and treatment requires more information about their biology, their roles in cancer dormancy, and immune evasion as well as in therapy resistance. Indeed, CTC functional and biochemical phenotypes have been only partially characterized using murine metastasis models and liquid biopsy in human patients. CTC detection, characterization, and enumeration represent a promising tool for tailoring the management of each patient with cancer. The comprehensive understanding of CTCs will provide more opportunities to determine their clinical utility. This review provides much-needed insights into this dynamic field of translational cancer research.

## 1. Introduction

**Figure 1:**
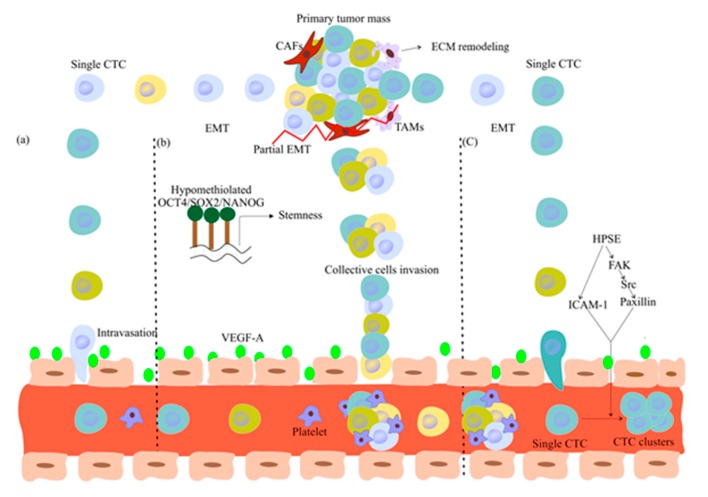
Models of CTC intravasation and cluster formation. (**a**) EMT allows single CTCs to detach from the primary tumor lesion; then, VEGF expression increases endothelial permeability and promotes CTC intravasation and dissemination to distant organs. (**b**) TAMs contribute to metastasis formation by ECM remodeling, and CAFs physically pull away cancer cells from the primary tumor. Partial EMT and stemness features due to *OCT4, NANOG, SOX2* upregulation in cancer cells within clusters enhance cell–cell junction formation and facilitate collective invasion, intravasation, dissemination, and metastasis initiation. (**c**) Clusters may be formed by intravascular aggregation of single CTCs that overexpress heparanase (HPSE) via HPSE-mediated induction of cell–cell adhesion molecules, such as intercellular adhesion molecule-1 (ICAM-1) and the focal adhesion kinase (FAK)-Src-paxillin pathway. Abbreviations: CTC, circulating tumor cell; EMT, epithelial-to-mesenchymal transition; VEGF, vascular endothelial growth factor; TAM, tumor-associated macrophages; ECM, extra cellular matrix; CAF, cancer-associated fibroblasts.

For metastasis formation, cancer cells must leave the primary tumor and disseminate. To this aim, epithelial tumor cells might go through epithelial-to-mesenchymal transition (EMT), lose their polarity and cell-cell/matrix adhesion, secrete specific enzymes (e.g., cathepsin D) to digest the extracellular matrix (ECM), and gain migratory properties [1,2] (Figure 1a). Then, invasive tumor cells reach the bloodstream to become circulating tumor cells (CTCs). The most aggressive CTCs might be the origin of metastatic tumors in distant organs via different mechanisms [3,4]. After extravasation, CTCs can invade a new organ and undergo mesenchymal-to-epithelial transition (MET). This phenotypic modification supports their proliferation as epithelial metastatic deposits or disseminated tumor cells (DTCs) [2,5]. Hence, tumor recurrence and metastasis formation can be attributed to cancer cell persistence and their dissemination in distant organs in patients without any clinical sign of metastatic disease [i.e., minimal residual disease (MRD)] [6]. 

During the last two decades, CTC detection, characterization, and enumeration opened a promising avenue to better understand the biology of metastatic cancer at the exact moment of metastasis initiation. Their use as a real-time “liquid biopsy” might help to predict metastasis formation and to develop novel anticancer therapies [6]. However, CTC clinical utility has not been demonstrated yet. Here, we discuss specifically three topics: (1) CTC biological features, (2) challenges and opportunities of CTC technologies, and (3) CTC potential clinical applications (e.g., diagnosis, prognosis, monitoring, and targeted therapy of solid cancers).

## 2. CTC Biology and Tumor Metastases

### 2.1. Do CTCs Have Cancer Stem Cell Features?

There is substantial evidence that many cancers are driven by cancer stem cells (CSCs) or tumor-initiating cells that are called metastasis-initiating cells (MICs) at metastatic sites. CSCs are a cancer cell population with stem cell features, such as self-renewal and differentiation into multiple cell types [7]. Stem cell-like CTCs, which are called circulating tumor stem cells (CTSCs), express putative stem cell markers, such as aldehyde dehydrogenase 1 (ALDH1) [8], DLG7 and BMI1 [9], CD44, and CD133 [10,11]. Moreover, epithelial cell adhesion molecule (EpCAM), which is an epithelial marker, has been detected in CTSCs with cytogenetic abnormalities [12]. Like CSCs, CTSCs go through a dynamic conversion upon exposure to environmental stress [13]. EMT, which contributes to tumor cell dissemination, also promotes cancer cell ability to self-renew [2,14]. Indeed, during EMT, acquisition of stem cell-like characteristics can provide survival advantages to cells that will contribute to the formation of distant metastases [15]. Stem cell-like and EMT-positive CTCs display higher invasion and migratory potentials, and also resistance to anticancer therapies [16]. Moreover, following targeted therapy of colorectal cancer, CTCs with self-renewal ability act as MICs causing tumor relapse [17].

It is not clear whether CTCs are representative of the whole tumor cell population or, similarly to CSCs, are a rare subpopulation [13]. Moreover, some migrating CSCs might give rise to CTCs that form metastases [11]. Like DTCs, chemotherapy-resistant CSCs might act as MICs that will lead to more aggressive tumors because of the selection pressures introduced by the anticancer treatment [15]. DTCs might be metastatic stem cells that can enter dormancy, thus contributing to MRD and acting as a source of cancer recurrence [15].

Genetic analyses have revealed similar mutation profiles in primary and metastatic tumors and also in the corresponding CTCs [18]. Nevertheless, the detection of unique mutations in CTCs but not in other tumor cells [18] suggests that CTCs share some features of CSCs, which sometimes are genetically distinct from the other tumor cells [15]. In addition, depending on the mutation frequency in CTCs, different stem cell-like features might be induced [19]. Overall, the acquired genetic and epigenetic alterations (i.e., clonal selection), mesenchymal phenotype, clustering, and tumor microenvironment-associated clues (e.g., hypoxia) seem to induce stem cell-like features in CTCs [15]. These findings suggest that CTCs with CSC and MIC features more easily reach and colonize distant organs and subsequently induce metastasis formation. 

### 2.2. Are Single CTCs or CTC Clusters Involved in Metastasis Formation?

The hypothesis that single CTCs must undergo EMT for metastasis initiation (Figure 1a) has been challenged by the finding that tumor cells break off from the tumor and travel in CTC clusters [20] (i.e., groups of >2 CTCs and up to large micro-emboli) [21]. CTC cluster role in metastasis formation has been validated by the molecular similarities of primary small clusters and of metastatic masses in human tumors [22]. Moreover, the finding that CTCs express epithelial markers also suggests that passive intravasation does not require EMT [23,24]. Indeed, it has been shown that CTC clusters escape from the primary tumor by collective invasion and detachment of small cell clusters [22]. 

Collective cancer cell invasion might be explained by two mechanisms. First, during embryo development and wound-healing, EMT leads to collective migration of neural crest cells [25] and cell sheets [26], respectively. Analysis of CTC clusters from squamous cell carcinoma revealed that Snail, an EMT transcription factor, induces the expression of the tight junctional protein claudin-11, promoting the formation of stable cell-cell contacts within the clusters for collective migration and invasion [27]. Therefore, partial EMT might enhance cell–cell junction formation in CTC clusters to elicit their collective migration and invasion (Figure 1b). Moreover, the increased hypomethylation of *OCT4, NANOG, SOX2* binding sites in CTC clusters enhances their stemness features and the expression of cell–cell junction components, thus promoting collective migration and invasion [20] (Figure 1b). Besides stemness, CTC clusters express the cell-surface vimentin (CSV) protein, an EMT marker that induces migration and survival signaling pathways in invasive tumor cells [28,29,30]. CTC clusters can contain actively proliferating and quiescent tumor cells, and also endothelial cells, cancer-associated fibroblasts (CAFs), and tumor-associated macrophages (TAMs) [31] (Figure 1b). CAFs provide survival and growth advantages after seeding at distant sites [32]. TAMs promote tumor cell invasion and consequently tumor progression [31]. They also secrete interferon molecules (IFN) that induce overexpression of bone marrow stromal antigen 2 (BST-2), a type II transmembrane protein that supports metastasis formation by mediating CTC survival, invasion, and dissemination [33]. Overall, CTC dynamic state between epithelial or mesenchymal states [1,29] and the heterotypic interactions among the different cell types within a cluster are critical for their survival and collective invasion [34].

Second, intravascular aggregation of single CTCs might cause the formation of clusters in the blood circulation [35] (Figure 1c). However, Aceto et al. suggested that CTC clusters are not formed in the blood circulation, and that CTCs break off from the primary tumor mass directly as clusters [36]. This hypothesis is supported by multicolor lineage-tracing experiments in a mouse model of pancreatic cancer [37]. Moreover, intravascular CTC aggregation might be inhibited by the shear forces generated in the bloodstream [38].

MET also is necessary for cluster cell seeding, as indicated by the expression of epithelial cytoskeletal keratin 5 (K5), K8, K14, and also of E-cadherin and P-cadherin (adhesion molecules) in disseminating cell clusters [34,39,40]. The overexpression of these epithelial markers facilitates the collective dissemination of cell clusters to colonize distant organs [34]. Moreover, the higher ability of clusters to seed and form metastases can also be attributed to overexpression of plakoglobin that promotes cell cluster integrity and survival upon reaching distant organs [36].

It has been reported that the detection of rare CTC clusters indicates a higher metastatic potential compared with high numbers of single CTCs [40]. This potential can be evaluated by assessing prognostic predictive indicators, such as overall survival (OS) and progression-free survival (PFS). The risk of disease progression is much higher in patients with many CTC clusters compared with patients with ≥5 single CTCs, but without any CTC cluster [41]. Moreover, the presence of large CTC clusters has been associated with poor OS in patients with metastatic breast cancer [41]. Similarly, prognosis is worse in patients with HER2-positive breast cancer with single CTCs and CTC clusters compared with patients in whom CTC clusters could not be detected [42]. However, OS after systemic therapy was significantly reduced in patients with CTC clusters [42]. Similarly, OS and PFS are reduced in patients with pancreatic ductal adenocarcinoma (PDAC) with many CTC clusters (≥30 clusters per 2 mL of blood) [43].

Polyclonal metastases might originate from the accumulation of multiple single CTCs or by direct seeding of a CTC cluster at a single site [40]. Several studies [36,40,44] demonstrated that polyclonal metastases arise from seeding of CTC clusters that contain polyclonal cells with higher survival and colony-forming potential. In conclusion, it seems that partial EMT/MET, stemness, ECM components, and cluster-associated cells are all involved and contribute to metastasis formation. 

### 2.3. CTC Dissemination and Dormancy

**Figure 2:**
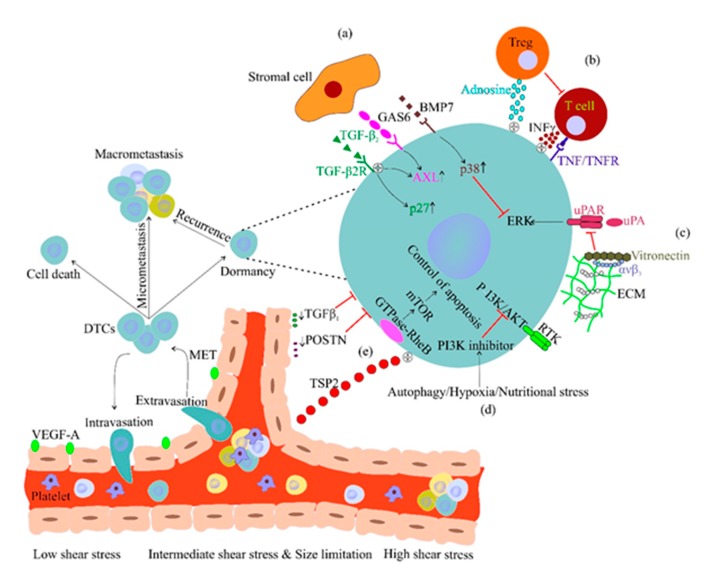
Cancer cell dormancy. Low shear stress and size limitation at vascular branches promote CTC extravasation. Disseminated tumor cells (DTC) dormancy is supported through different mechanisms: (**a**) Growth arrest-specific protein 6 (GAS6) activates *AXL*-associated dormancy signaling. Transforming growth factor-β2 (TGFβ2) and BMP7 signaling mediate the production of cell cycle inhibitors by dormant cells. (**b**) Regulatory T (Treg) cells-derived adenosine and tumor necrosis factor receptor 1 (TNFR1) signaling protect tumor cells against oxidative stress and induce dormancy signals, respectively. (**c**) The ECM integrin αvβ3 binds to urokinase plasminogen activator (uPA), and suppresses the dormancy effect of Extracellular signal-Regulated Kinase (ERK) signaling. (**d**) Stressful conditions result in PI3K-AKT survival pathway inhibition. (**e**) The GTPase RHEB-mTOR pathway suppresses apoptosis in DTCs. Endothelial cells produce thrombospondin 2 (TSP2) that maintains DTCs in a dormant state; however, TGFβ1 and periostin (POSTN) promote DTC proliferation.

Once in circulation, CTCs are stopped at branch points in vessels due to low shear stress and size limitations (CTCs up to 20 μm in diameter versus capillaries of ~3–7 μm) (Figure 2). Then, CTC-/immune cell-secreted enzymes, such as matrix metalloproteinases (MMPs), and vascular endothelial growth factor (VEGF) increase endothelial permeability to allow CTC extravasation [45]. Exosomes also have a role. These small extracellular vesicles regulate cell–cell communication by horizontal transfer of biomolecules, and they express integrins at their surface that mediate site-specific metastasis formation. For example, exosomes that express the α6β4 and αvβ5 integrins are associated with lung and liver metastases, respectively. Moreover, uptake of such integrins by resident cells leads to activation of the pro-migratory and pro-inflammatory S100 gene to prepare the pre-metastatic site, thus promoting CTC homing at the metastatic niche [46]. Moreover, exosome uptake by resident cells results in the release of CTC-supporting components [46].

Although lymphatic vessels can uptake only particles of ~5–300 nm in diameter [47], melanoma metastasis formation is supported via lymphangiogenesis. Olmeda et al. found that primary melanoma cells secrete exosomes that contain the growth factor midkine. Midkine-positive exosomes circulate to pre-metastatic lymph nodes in distant organs where midkine activates mammalian target of rapamycin (mTOR) signaling to increase the expression of VEGF-R3, through which major lymphangiogenic signals are transduced and the sites are prepared for CTC arrival [48].

Aggressive tumors release thousands of cancer cells in the circulation each day; however, the metastasis rate is very low [5] and it is a very inefficient process [49]. Metastasis formation and progression depends on CTC cluster behavior during the different metastatic cascade steps, and on their protection from hemodynamic stress [2]. Then, cancer cells need to adapt to the new microenvironment where they are exposed to interactions with stroma cell types, diffusible signals and ECM proteins [5], differentiation and growth-inhibiting signals [50], interactions with resident niche cells [51], and deleterious signals [52]. 

Moreover, depending on their origin (late-stage primary tumor or pre-invasive lesion), DTCs may give rise to metastatic lesions or enter dormancy, respectively [53]. Cancer cell dormancy is a reversible arrest in the G0/G1 phase of the cell cycle. Lack of growth-promoting signals, ECM cooperation, stromal cells, immune system and vascular niches support DTC survival and dormancy at metastatic sites [32] (Figure 2a–e). 

Production by bone stromal osteocytes of the ligands for tyrosine kinase receptors, such as growth arrest-specific protein 6 (GAS6), bone morphogenetic protein 4 (BMP7), and transforming growth factor-β2 (TGFβ2), support DTC dormancy (Figure 2a). GAS6 activates *AXL* signaling that is associated with quiescence of disseminated cells [54]. TGFβ2 induces the expression of the cell cycle inhibitor p27 in bone resident DTCs, promoting their quiescence [54]. BMP7 signaling activates p38 that stimulates the expression of cell cycle inhibitors [55]. Regulatory T (Treg) cells produce adenosine that promotes quiescence and protects resident cells from oxidative stress [56]. T cell-activated IFNγ and tumor necrosis factor receptor 1 (TNFR1) signaling act as dormancy-inducing signals in DTCs [57] (Figure 2b). As CD8+ T cells kill cycling DTCs, quiescent DTCs are protected from T cell cytotoxic effects [58]. 

Binding of urokinase plasminogen activator (uPA) to its receptor mediates Extracellular signal-Regulated Kinase (ERK) activation and cell proliferation that is then reduced by binding of the ECM integrin αvβ3 to vitronectin, resulting in uPA decrease [59] (Figure 2c). Loss of endothelium adhesion and nutritional or hypoxia stress lead to secretion of PI3K-AKT pathway inhibitors in DTCs, resulting in quiescence and induction of autophagy to maintain their metabolic fitness [60,61,62] (Figure 2d). Upregulation of the GTPase RHEB induces mTOR activation that protects DTCs from apoptosis [63]. Finally, thrombospondin 2 (TSP2) secreted by endothelial cells maintains solitary DTCs in a dormant state, while TGFβ1 and periostin (POSTN) promote dormancy exit, leading to an increase in tumor size and recurrence [64] (Figure 2e).

### 2.4. How CTCs Escape the Immune System Surveillance and Therapy?

**Figure 3:**
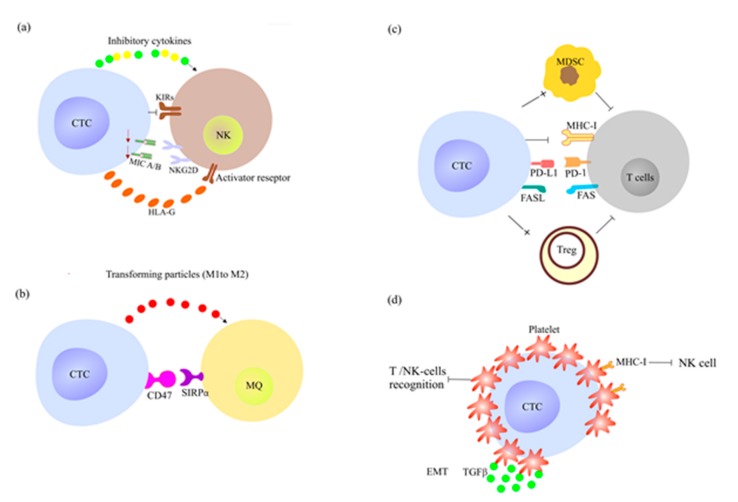
CTCs evade the immune systems in several ways (**a**–**d**). (**a**) CTCs modulate NK cell activity by producing inhibitory cytokines and blocking NK cell activator receptors (e.g., NKG2D and KIRs). (**b**) CTCs induce production of tumor-supporting M2 type of macrophages (MQ). M2 macrophages induce matrix-remodeling, neoangiogenesis, and exert an immune suppressive phenotype, which all support tumor metastases [78]. Binding of CTC CD47 to macrophage SIRPα inhibits tumor cell phagocytosis by macrophages. (**c**) PDL-1 and Fas Cell Surface Death Receptor Ligand (FASL) upregulation at the CTC surface reduces T-cell anti-tumor responses and induces T-cell apoptosis, respectively. CTCs exploit Treg cells and myeloid-derived suppressor cells (MDSCs) to promote metastasis formation in patients with various malignancies. (**d**) Platelets interact with CTCs, and protect them from antigen recognition by immune cells. Platelets produce MHC-I-positive vesicles, thus sending self-signal to NK cells. Platelets produce also TGF-β, a factor that initiates and maintains EMT, and helps CTCs to evade immune attacks [79]. Abbreviations: NKG2D, NK cell receptor D; SIRPα, signal-regulatory protein α; PD-L1, programmed death ligand 1; Treg, regulatory T cells.

The immune system should eliminate or at least control CTC activity [65]. The interactions between immune cells and tumor cells reach a dynamic equilibrium to restrain tumor development. However, clonal selection can lead to CTC immune escape [66] through suppression of the anti-tumor immune responses (Figure 3).

#### 2.4.1. CTCs Escape the Innate Immune System Response

During metastasis formation, the overall natural killer (NK) cell count is increased. CTCs can modulate NK cell anti-tumor activity through production of inhibitory cytokines (e.g., IL-1β, IL-8, IL-10, and prostaglandin E2) that block the NK cell immunoglobulin receptors (KIRs) [2,3] (Figure 3a). Toll-like receptors (TLRs), which are expressed on macrophages and dendritic cells (DCs), can activate NK cell cytolytic killing [67]. The increase of CTC number in metastatic cancers is associated with TLR down-regulation and NK cell activity inhibition [67]. Moreover, CTCs also reduce the therapeutic efficacy of adoptive transfer of autologous NK cells after chemotherapy [62].

The major histocompatibility complex (MHC) is a set of cell surface proteins that helps to recognize viral and tumor-associated antigens for initiation of the immune cell response [68]. NK cell receptor D (NKG2D) recognizes MHC class I polypeptide-related sequence A (MICA)/MICB on neoplastic cells [69,70]. Oncogenic microRNAs and upregulated hypoxia-inducible factor 1α (HIF-1α) mediate MICA/MICB downregulation and elimination on stem-like breast cancer cells, respectively, thus reducing NK cell activity [69,70]. Also, MHC I downregulation on metastatic breast and colon CTCs reduces NK cell anti-tumor activity, and correlates with higher tumor grade and propensity for regional lymph node metastases [68]. The correlation of unfavorable clinical outcomes with high expression of vesicle-bound soluble HLA-G (sHLA-G) and the presence of stem-cell like CTCs in patients with breast cancer after neo-adjuvant chemotherapy indicates that CTCs exploit sHLA-G to escape NK cell-mediated cytotoxicity [71]. Indeed, HLA-G expressed on malignant cells blocks immune cell receptors, such as KIRs and CD8, and thus suppresses NK and T-cell cytolytic activity [72].

The high shear forces in lung capillaries induce micro-particle production by CTCs. Following their uptake and ingestion, macrophages are activated and contribute to metastatic lesion initiation and development [67]. In physiological conditions, CD47 (the ‘do not eat me’ signal) at the leukocyte surface binds to signal-regulatory protein α (SIRPα), its ligand on macrophages and DCs, inhibiting leukocyte phagocytosis [73]. CTCs can overcome their programmed removal through CD47 upregulation at their surface [74] (Figure 3b).

The characterization of individual white blood cells (WBCs) in CTC clusters of breast tumor-bearing mice has shown the 85.5–91.7% of WBCs express neutrophil markers [75]. These clusters overexpress neutrophil stimulator cytokines (e.g., CSF1, TGF-β3, and IL-15) and cluster-forming markers, such as F11R, ICAM1, ITGB2, and VCAM1, that collectively show a collaborative behavior toward tumor metastasis [75]. Binding of ICAM-1 and L-selectin expressed by CTCs to MAC-1/β2-integrins and CD44v on neutrophils, respectively, leads to neutrophils interaction with CTCs for adhesion to the pulmonary endothelium and extravasation [76,77]. After extravasation, neutrophils induce cell cycle progression and DNA replication in CTC clusters, promoting CTC survival and proliferation and consequently favoring metastasis seeding [75].

#### 2.4.2. CTCs Escape the Adoptive Immune System Response

CTCs downregulate MHC-I expression on CD8 T cells, a receptor that is crucial for initiation of the adaptive cytotoxic T lymphocyte response [2]. Programmed death ligand 1 (PD-L1) is the immune-checkpoint molecule that binds to PD-1 in self-reactive T cells and induces immune tolerance to prevent autoimmune responses [80]. In patients with breast and ovarian cancer, tumor cells evade the adoptive immune system by expressing PD-L1 that suppresses CD8+ T-cell anti-tumor activity [10]. PD-L1 expression on CTCs isolated from patients with human epidermal growth factor receptor 2 (HER2)-negative breast cancer suggests that these tumor cells can escape the immune system [81] (Figure 3c). PD-L1 upregulation on CTCs correlates with high EMT and CD44+ cell level in patients with primary head-and-neck squamous cell carcinoma [82,83]. 

Fas Cell Surface Death Receptor (FAS or CD95) belongs to the TNF transmembrane receptor superfamily. After binding to its ligand Fas Cell Surface Death Receptor Ligand (FASL), FAS can activate the extrinsic apoptosis pathway through recruitment of caspase 8 and 10, and consequently mediates cells death. In resistant tumor cells, FAS also induces non-apoptotic signaling pathways linked to tumor growth, survival, and migration [84]. FASL on tumor cells bind to FAS receptors on T cells and initiates T-cell apoptosis [85] (Figure 3c). The high frequency of FASL+ CTCs or Treg cells and FAS+/CD8+ cytotoxic T or CD4+ T-helper cells in patients with breast cancer indicates that CTCs hinder the anti-tumor immune response through the FAS/FASL apoptotic pathway [85,86]. Moreover, CTCs promote the presence of tumor-supportive immune cells, such as myeloid-derived suppressor cells (MDSCs) and Treg cells (Figure 3c). MDSCs and Treg cells increase the circulating levels of IL-1β, IFNγ, and CXCL10 [87,88] that suppress the peripheral anti-tumor immune responses, and support CTC survival [89,90]. In a mouse model of colorectal cancer, CTC frequency was positively correlated with the serum levels of IL-17A, a pro-inflammatory cytokine. Indeed, IL-17A-secreting CD4+ T cells induce MMP-9 expression in CTCs that consequently promotes their extravasation at distant organs [89].

CTCs modulate the immune system also by increasing platelet number and activity [79] (Figure 3d). Platelet–CTC interaction hinders CTC recognition by NK cells and T cells [90], reduces the shear stress experienced by CTCs in the bloodstream, and increases CTC extravasation in a selectin-dependent process [85]. CTCs evade NK cell cytotoxicity by integrating their own cell membrane with platelet-derived MHC-I-positive vesicles. In this way, CTCs acquire a ‘normal’ platelet phenotype and can signal to NK cells: “Do not harm the self” [91]. Platelets also support CTC proliferation, and contribute to the formation of their bone niche by producing IL-6- and IL-8 that induce osteoclast activity [92]. Overall, these findings should help develop novel anticancer immunotherapies that specifically target key actors in the metastatic process.

#### 2.4.3. CTCs Induce Resistance to Chemotherapy

The major mechanisms of chemotherapy resistance in tumor cells and CSCs [7] have also been observed in CTCs. Klameth et al. revealed that CTCs from patients with small cell lung cancer exploit mechanisms of chemotherapy resistance and survival, such as (a) increasing drug efflux, (b) target inactivation or mutation, (c) overexpression of the quiescence marker p27 (Kip1), (d) downregulation of the proliferative marker Ki67, (e) suppression of oxygen radical production, and (e) increasing genomic DNA repair [93]. Similarly, in CTCs from patients with endocrine-resistant metastatic breast cancer, estrogen receptor (ER) and B-cell lymphoma-2 (BCL-2) are downregulated, whereas Ki67 and HER2 are upregulated [94].

Moreover, EMT and dormancy mediate the upregulation of ECM remodeling markers and the downregulation of signaling pathways that induce tumor cell mitosis [92,95,96]. The increase of tumor recurrence and resistance correlates with the presence of CTCs that underwent EMT [97]. The EMT phenotype also enhances CTC mesenchymal and stem-like phenotypes that promote their dormancy and also recurrence after therapy [96]. Overexpression of plastin 3 and CSV in EMT-positive CTCs from patients with metastatic colorectal and breast cancers promotes CTC drug-resistance and dormancy [98,99]. 

Finally, hypoxia stress signals ensure CTC survival through activation of autophagy to provide the nutrients (e.g., amino acid, ATP, fatty acid) required for cell metabolism [27]. Immune cells express TNF-related apoptosis-inducing ligand (TRAIL) that binds to the tumor cell membrane death receptors 4/5 (DR4/5) and induces the extrinsic caspase-dependent apoptosis signaling pathway in tumor cells [3,100]. However, autophagy induces DR4/5 endocytosis on CTCs surface, resulting in their degradation and resistance to TRAIL-mediated apoptosis [27]. 

To conclude, as CTCs contribute to promoting resistance to conventional anticancer treatments, investigating and targeting these mechanisms represent a potential therapeutic approach.

## 3. CTC Enrichment Strategies

**Figure 4:**
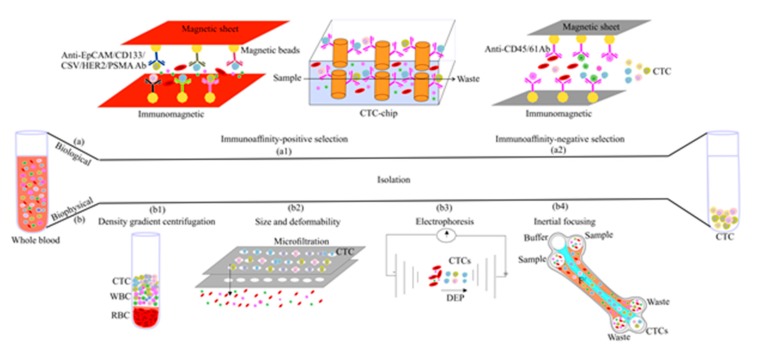
Methods for CTC enrichment based on their (**a**) biological, and (**b**) biophysical properties. Immunoaffinity-based CTC isolation using two strategies: (**a1**) Positive enrichment where CTCs or CTSCs are captured by immunomagnetic devices or CTC-chips by trapping their surface markers, such as EpCAM, CD133, HER2, CSV, prostate-specific membrane antigen (PSMA); and (**a2**) Negative enrichment by depletion of WBCs and platelets using anti-CD45 and -CD61 antibodies, respectively. (**b1**) Density: CTC density is lower than that of red blood cells and white blood cells; (**b2**) Size and deformability: filters with pores of 7- 8µm in diameter allow the passage of blood components, but not of CTCs; (**b3**) The distinct electrical charges of CTCs and blood cells allow separating these cell types; and (**b4**) Inertial focusing: CTCs are passively separated using microfluidic devices based on their size through the application of inertial forces (shear-gradient lift force and a wall effect lift force) that affect cell positioning within the flow channel.

Currently, CTCs are considered a promising bio-source for cancer detection. Many technologies have been and are developed for CTC capture and isolation based on their biophysical and biological features [6] (Figure 4). The challenges and opportunities of CTC enrichment are summarized in Table 1.

### 3.1. Biological Feature-Based CTC Enrichment

Biological feature-based CTC enrichment is usually performed using immunoaffinity approaches based on CTC trapping or removal of background blood cells (positive and negative selection, respectively) (Figure 4a) (Table 1). 

#### 3.1.1. Positive Enrichment

Immunomagnetic devices and microfluidic chips are two examples of positive enrichment technologies in which monoclonal antibodies (mAbs) targeting CTC surface antigens are coupled to magnetic beads or micro-posts/surfaces, respectively (Figure 4a1). For instance, the CellSearch^®^ (Menarini Silicon Biosystems, Inc.; Bologna, Italy) system is an immunomagnetic device based on EpCAM-positive CTC enrichment that offers semi-automated CTC capturing, staining, and image analysis, and thus can overcome the problems of CTC stability during sample shipment and storage [99,101]. Affinity-based microfluidic devices are divided in micro-post-based methods, which require chemical modifications for mAbs coating, and surface-based devices that use antibody-coated surfaces. For instance, CTC-Chip includes 78,000 micro-posts that are chemically functionalized with an anti-EpCAM antibody for CTC capture [102]. In this system, the micro-post geometric arrangement and the precisely controlled fluid flow velocity and direction enhance the capture of viable CTCs in pre-processed blood and whole blood from patients with different cancers [103,104]; however, false-positive results show how difficult it is to detect CTCs [103]. Moreover, in surface-capture devices (e.g., GO chip, GEM chip, and HB chip), CTCs are released from the device surface by trypsinization and this may cause loss of the receptors expressed at the CTC surface [103]. This problem has been solved by designing smaller microfluidic chips with magnetic pores and using vertical flow centrifugation for the release of intact CTCs [105]. Aptamers also represent a good alternative to antibodies due to their high affinity, low cost, easy modification, and simple release mechanisms [106].

However, cancer cells undergoing EMT may lose EpCAM expression [99]. To overcome this limitation, alternative strategies, such as EMT-positive CTC enumeration based on CSV expression, have been developed [99]. As CSV is widely expressed in WBCs, results need to be confirmed by additional analyses, such as fluorescence in situ hybridization (FISH) that can detect the tumor-specific genomic changes [107]. Another alternative strategy is the enumeration of EMT-like CTCs based on CSV and CD133 expression. This technique showed that CSV+ CD133− tumor cells have an EMT phenotype (i.e., express E-cadherin, Twist and Slug), and they act as CTSCs or metastatic CSCs, and also exhibit higher metastastatic properties compared with CSV- CD133+ cells [108]. Moreover, in patient with glioblastoma multiforme, CSCs that co-express CSV and CD133 display tumor initiating cell features and aggressive properties [109]. 

CTC affinity selection (and thus CTC purity) is influenced by the surface mAb concentration, and low antigen level leads to mixed monolayers and recovery reduction. Moreover, some antibodies target only specific cancer types (e.g., PSMA-, HER2-expressing tumors) or lack cancer-specificity because their targets are expressed also by other cells (e.g., CD133, VCAM-1, ICAM-1) [110]. These limitations might be overcome by using dual CTC selection. Fibroblast activation protein alpha (FAPα) is expressed in most human epithelial cancers and has been associated with mesenchymal features [110]. Dual selection with anti-FAPα and -EpCAM antibodies provides high positivity and specificity, and might be used to monitor and to obtain precious information on resistance to combination therapies and on cancer progression [110]. The co-expression of EpCAM and CD133 in lung CSCs highlights their potential for tumor metastasis progression [111]. In patients with PDAC, a microfluidic platform functionalized with mAbs against EpCAM and CD133 allowed showing that the majority of isolated cells are CTCs (84.4%) and CSCs (70.8%) [112]. Using cocktails of different mAbs is critical to increase CTC yield, as done in the AdnaTest assay that uses a cocktail of anti-EpCAM, -MUC-1, -HER2, and AdnaTest Breast mAbs for capturing CTCs in blood from patients with solid tumors [113]. 

Centrifugation or reed blood cells (RBCs) lysis as pre-processing steps can lead to CTC loss. This problem can be addressed by using another immunomagnetic enrichment technology (i.e., MagSweeper) that captures CTCs from whole diluted blood samples pre-labeled with magnetic particles by bead-bound CTC absorption using magnetic rods [114].

To overcome the issues of CTC low number in blood samples and of CTC stability during sample shipment and storage, leukapheresis and cytopheresis are an excellent option for increasing the chance of ex vivo CTC capture and detection of rare CTCs. During cytopheresis, blood is passed through a machine that retain CTCs, while WBCs and peripheral blood stem cells go back to the patient’s vein [115]. Standard cytopheresis is an invasive and time-consuming method and this hampers routine clinical use [107]. Novel cytopheresis devices can be placed in the antecubital vein of a patient to allow the in vivo binding of rare EpCAM-positive CTCs from the circulating blood [116,117]. However, their repeated application is limited due to the possible systemic exposure to excess iron caused by the pre-injection of magnetic particles or wires [118]. To this aim, Kim et al. developed a herringbone graphene oxide (HBGO) CTC chip coated with anti-EpCAM mAbs that allows capturing CTCs from circulating blood in about 2 hours [118].

#### 3.1.2. Negative Enrichment

Positive enrichment has other shortcomings. Indeed, some CTCs are not captured by the difficult-to-remove antibodies, and the capture accuracy is reduced by the heterogeneous expression of cell surface biomarkers due to EMT [104]. Moreover, circulating EpCAM+ cells appear in other diseases, such as benign colon disease, and can be confused with CTCs [119]. These pitfalls can be overcome by label-free methods (see Section 3.2), or negative selection in which labeled-magnetic beads or bi-specific antibodies are used to deplete hematopoietic CD45+ leukocytes and CD61+ platelets cells [120] (Figure 4a2). However, with this method, another step is needed for the isolation of circulating CD45– endothelial cells [120].

Overall, while immunoaffinity-based CTC enrichment presents valuable advantages, it also has some limitations. In label-dependent methods, antibody binding to surface markers causes the activation of intracellular signaling pathways [121], and the subsequent analysis of intracellular signaling in the isolated CTCs might result in artifacts. Therefore, surface label-free methods may be a better choice if the downstream analysis (i.e., transcriptome or epigenomic modification) of CTCs is required. Additionally, CTC dynamic state leads to phenotypic heterogeneity [1,29], thus complicating their identification and selection.

### 3.2. Physical Feature-Based CTC Enrichment

CTC enrichment methods based on biophysical properties, also called “label-free” approaches, can isolate CTCs from blood by exploiting their specific density, size, deformability, and electric charges (Figure 4b) (Table 1). 

#### 3.2.1. Density-Based Enrichment

Density-based CTC enrichment technologies, such as Ficoll-Paque^®^ (GE Healthcare, Chicago, USA) use density gradient centrifugation (Figure 4b1) in which centrifugal forces separate blood cells based on their sedimentation rate that is determined by the cell size and density. RBCs and neutrophils have higher buoyant densities, unlike other mononuclear lymphocytes and CTCs. Thus, CTCs remain in the upper layer, allowing their collection and analyses [122]. However, centrifugation systems might lead to significant CTC loss, especially high-density CTCs. This issue can be solved by label-free microfluidic technologies and/or by the OncoQuick^®^ (Greiner Bio-One, Frickenhausen, Germany) gradient system that leads to higher CTC enrichment compared with the standard Ficoll-Paque^®^ method [120].

#### 3.2.2. Size-Based Enrichment

Size-based enrichment uses microfluidic devices, such as 2D membrane microfilters (e.g., ISET^®^, ScreenCell^®^, and CellSieve™) and 3D membrane microfilters (e.g., FaCTChecker, Parsortix system, and Resettable Cell Trap), to isolate single CTCs or CTC clusters based on their size and/or deformability (e.g., Cluster-Chip) (reviewed by Ferreira et al. [104]) (Figure 4b2). Cross-chip is a label-free microfluidic cell filter that directly isolates CTCs from unprocessed whole blood samples based on their size and deformability with high efficiency and purity, and then characterizes them by phenotypical and molecular analysis (e.g., droplet digital PCR) [122]. Although CTCs (12–25 μm) are generally larger than WBCs (8–14 μm), false-positive results are observed due to CTC variable diameter during clustering, apoptosis, and the different stages of the cell cycle [104,123]. Size-based isolation can be improved by eliminating normal cells in the downstream analysis, such as array-Comparative genomic hybridization (CGH) [123]. 

Traditional isolation technologies cannot recover intact clusters with high sensitivity and specificity [107]. For instance, the reduction of CTC surface-to-volume ratio upon clustering reduces the efficiency of antibody-based platforms to detect large clusters [106]. Integrating flow cytometry-based isolation of single CTCs and clusters with whole transcriptome analysis allowed detecting one viable tumor cell in one million WBCs in a pancreatic cancer mouse model [124]. However, flow cytometry can damage CTC clusters and requires long residence times [31,125]. To address these limitations, two-stage continuous microfluidic chips, based on cluster size and their inherent asymmetry, have been developed for the recovery of clusters from whole blood with minimal cluster dissociation and maximal viability [125]. The effect of filtration on cluster integrity can be reduced with a microfluidic chip approach recently described by Edd et al. in which a microscope slide-sized PDMS device is used to isolate untouched CTC clusters from pre-concentrated samples obtained from large blood volumes with 80% efficiency [126].

#### 3.2.3. Dielectrophoresis

Dielectrophoresis (DEP) is exploited by the DEPArray system for selection and isolation of single CTCs, independently of their features, followed by massive parallel sequencing to clearly reveal differences among CTCs [127]. In this system, the cell composition, morphology and phenotype lead to different DEP migration and retention between CTCs and other cells, thus allowing their isolation (Figure 4b3). 

#### 3.2.4. Inertial Focusing

CTCs can be isolated with high purity using hydrodynamic microfluidic devices based on inertial forces that force blood cells to migrate across flow stream lines to equilibrium positions (Figure 4b4). For instance, the new inertial multi-flow microfluidic/microchannel (MFM) device separates CTCs in blood samples after RBC pre-lysis by exploiting their size-dependent inertial migration (label-free method). MFM can isolate CTCs from blood samples of patients with non-small-cell lung cancer (NSCLC) with >99% of efficiency and >87% of purity [128]. Sometimes, microfluidic devices show low sensitivity and reproducibility (due to size overlap between CTCs and WBCs). This limitation was recently addressed by a device made of micro-ellipse filters with different arrays of 12 micro-filters that can reliably capture viable CTCs with high purity and efficiency without hydrodynamic forces [129]. 

Zeinali et al. developed the inertial microfluidic Labyrinth device that can isolate single CTCs and CTC clusters (100% and 96% of cell recovery, respectively) from blood samples of patients with NSCLC using a high-throughput, biomarker-independent, and size-based (by inertial focusing and Dean flow) isolation method [130]. The authors reported that Labyrinth allowed the recovery of heterogeneous CTCs (higher number of EpCAM- and CSV+ CTCs than of EpCAM- cells). This method should allow assessing heterogeneous CTC/cluster subpopulations and also detecting the CTCs missed by traditional antibody-based capture methods. 

Other inertial focusing methods have been developed that can capture viable cells, for example ClearCell^®^ FX or the Vortex method, however these methods still require further validation [131,132]. 

### 3.3. Combined Methods

CTC enrichment methodologies have their own advantages and limitations (Table 1), and several clinical trials have assessed the clinical value of CTC testing (Table 2). Currently, the CellSearch^®^ system is the only US Food and Drug Administration (FDA) approved CTC detection system, and newly developed technologies are routinely compared with this system [104]. Unfortunately, CTC purification using immunoaffinity-based methods is too expensive for its broad implementation in clinical settings. Label free-based methods are inexpensive and better capture the heterogeneous nature of CTCs. However, biophysical-based CTC selection needs to be supported by molecular-based CTC detection. Microfluid systems have been commercialized, but such sophisticated systems need to be automated and this increases the cost of such devices, thus limiting their implementation [133]. 

One alternative approach is to combine the advantages of biophysical- and biological-based enrichment methods, such as geometrically enhanced differential immunocapture (GEDI; size and immunoaffinity selection), cell enrichment and extraction (OncoCEE; size and immunoaffinity selection and in situ staining), and RosetteSep™ (density centrifugation and immunoaffinity selection). For instance, the GEDI chip can increase (2–400-fold) CTC counts compared with CellSearch^®^ [104]. A microfluidic immunomagnetic-based CTC isolation device has been developed (CTC-iChip) that can sort epithelial and non-epithelial cancer cells using a label-dependent or a label-free process (micropillar array, hydrodynamic size-based sorting, and magnetophoresis) [16,103]. CTC-iChip can capture and culture CTCs for downstream characterization [16]. 

Besides CTC enrichment and enumeration, the detailed molecular and functional characterization of isolated CTCs can be performed using high-throughput single-cell analytical methods [134]. Moreover, in vitro culture of CTCs and in vivo models, such as CTC-based patient derived tumor xenografts and CTC-derived xenografts, combined with individual patient information can help to better understand cancer prognosis for personalized medicine [135]. Finally, CTC clusters from different metastatic sites have different phenotypes [107]. Tanaka et al. used a tissue-clearing protocol and 3D light-sheet microscopy, called immunolabeling-enabled 3D imaging of solvent-cleared organs (iDISCO), to analyze formalin-fixed paraffin-embedded samples [136]. This approach can identify unique patterns of phenotypic heterogeneity (EMT and angiogenesis) in solid tumors and is a promising strategy for the characterization of cluster heterogeneity.

### 3.4. Challenge with DTC Isolation 

While technologies are focused on CTCs in peripheral blood, before and during dissemination, DTCs that successfully land at distant organs, such as bone marrow and lymph nodes, can form recurrent tumors and metastases [5]. After successful treatment of the primary lesion, bone marrow may become a DTC reservoir, seeding them regularly in the circulation [5]. Bone marrow sampling for DTC analysis is more invasive than blood sampling and difficult to repeat [16]. Moreover, while CTCs are mostly detected and studied in patients with metastases, DTCs can be detected in bone marrow at early disease stages [15], questioning the time of metastasis initiation and CTC prognostic value in the peripheral blood at late cancer stages. 

## 4. CTC Clinical Relevance 

Tumor tissue biopsy and fine-needle aspiration are invasive procedures that allow sampling only part of the tumor mass. Therefore, they are not representative of the whole disease. Moreover, these procedures may lead to tumor cell seeding around the sampling area and increase the risk of local dissemination [157]. Hence, low or non-invasive methods are required to improve the early diagnosis of cancer that is crucial to reduce the mortality of this disease. As CTCs are released into the blood circulation already by relatively small and undetectable primary tumors [158], their analysis is considered a minimally/non-invasive liquid biopsy that can be performed repeatedly to provide real-time information on solid cancer progression. 

### 4.1. How CTCs Improve Metastasis Prediction and Diagnosis?

At diagnosis, CTC number is correlated with PFS and OS [6]. In early stage prostate [159], breast [160] and lung cancer [161], the presence of more than five CTCs per 7.5 mL of peripheral blood is a strong predictor of PFS reduction, whereas the detection of fewer than five CTCs per 7.5 mL is predictive of better OS. In gestational choriocarcinoma, patients with a CTC count ≥4 are at higher risk of distant multiple-organ metastases, and of chemotherapy resistance [162]. CTC count increases with tumor progression and development of distant metastases [163]. Analysis of CTCs in patients with testicular germ cell tumors showed that CTC were detected in 41% of patients with metastatic tumor and in 100% of patients with relapsed tumor [164]. The low accuracy of MRD monitoring based only on CTC count could be improved by investigating simultaneously CTCs in blood and DTCs from bone marrow, as done to predict disease-free survival (DFS) in patients with primary breast cancer. However, no significant association was observed between DTCs and CTCs [165]. 

In patients with breast cancer, the correlation between tumor size, CTC counts, and survival revealed that CTC number is closely associated with the primary tumor size, the number of metastases, and PFS reduction [166]. Conversely, CTC number was significantly higher in small than large primary hepatocellular carcinoma [158]. This discrepancy may be due to the intrinsic heterogeneity of small volume tumors and to peri-tumoral inflammation that facilitates CTC entry in the blood stream [158]. 

Balakrishnan et.al. studied the clinical implications of CTC clusters, and showed that in vitro cluster formation from CTCs of patients with advanced stage lung and breast cancer correlates with shorter OS. They also found that patients who were sensitive and resistant to chemotherapy exhibited loose and tight clusters, respectively. Moreover, tight clusters were clearly correlated with poor patient survival [167]. Conversely, in early-stage lung cancer, patients with CTC clusters exhibit significantly lower recurrence-free survival and OS than patients with single CTCs or undetectable CTCs [168].

CTCs may provide useful information for tumor staging at diagnosis. For instance, in gastric cancer, the percentage of patients with ≥4 CTCs increases with the tumor stage and reaches 95.24% at stage IV [162]. Moreover, CTC count ≥4 is associated with significantly higher risk of distant organ metastases [162]. In the clinic, liquid biopsy for CTC detection/enumeration should be performed particularly in the period after diagnosis of the primary tumor and before the detection of metastatic lesions (i.e., “metastatic latency”). 

### 4.2. Can CTC Profiling Guide the Therapeutic Strategy?

#### 4.2.1. CTC Proteomic Analysis 

Determining the tumor cell phenotypic status and its correlation with CTCs can help treatment decision-making [169]. In patients with breast cancers, the presence of HER2-positive CTCs is associated with poorer PFS compared with HER2-negative CTCs. Moreover, HER2-positive CTCs can be detected also in patients with HER2-negative primary tumors. Thus, CTC HER2 status could help to determine the appropriate treatment based on the HER2 profile [10]. Similarly, ER-positive primary tumors produce ER-negative CTCs in the metastatic phase, and their detection predicts resistance to endocrine therapy in 20% of patients with an ER-positive tumor [170]. High androgen receptor splice variant 7 (ARV7) mRNA or protein expression level in CTCs from patients with advanced prostate cancer is associated with resistance to therapy with enzalutamide and abiraterone to target androgen receptors [171]. Moreover, the simultaneous enrichment/detection and quantification of ARV7-positive CTCs in 7.5 mL of blood showed their correlation and predictive value for treatment response [172], thus guiding towards alternative therapies, for instance taxane [173], or metformin combined with enzalutamide as shown in a clinical trial on patients with metastatic castration-resistant prostate cancer (ClinicalTrials.gov ID: NCT0264053).

Analysis of PD-L1 expression in CTCs from patients with metastatic lung cancer before and after immunotherapy showed that the overall response to anti-PD-1 immunotherapy was higher in patients with >1.32 CTCs/mL and in those with >50% of PD-L1-positive CTCs [174]. Increased PD-L1 expression on CTCs (≥5% of PD-L1-positive CTCs) in patients with metastatic lung cancer after radiation therapy was associated with poor prognosis [175]. Moreover, PD-L1 overexpression in CSV+ CTCs has been associated with poor prognosis and poor OS in patients with colorectal cancer [176]. Thus, PD-L1-positive CTCs represent a candidate predictive or prognostic marker of malignancy and treatment response [177].

Finally, metastatic CSC subsets that express specific markers, such as CD44v6 in colorectal cancer [177], CD133 and CXCR4 in pancreatic cancer [178], and ALDH and CD44 (but not CD24) in breast cancer [179], are involved in metastasis formation in distant organs. Therefore, their identification in primary tumors will be essential for the diagnosis, and even the prevention of tumor metastases. CTC proteomic characterization based on these stem cell biomarkers is currently tested for therapy personalization.

#### 4.2.2. CTC Genomic Analysis

The genetic analysis of mutations in CTCs could improve cancer management. Analysis of PIK3CA mutations in single CTCs from 20 patients with metastatic breast cancer showed that six harbored a PIK3CA mutation that may help to select the best treatment [180]. Epidermal growth factor receptor gene (EGFR) mutational analysis in CTCs from 27 patients with metastatic lung cancer treated with tyrosine kinase inhibitors found the presence of the *EGFR-T790M* drug-resistance mutation in 92% of them. This was correlated with PFS reduction during treatment [181]. 

Single CTC mutation analysis highlighted the presence of mutations in genes encoding the therapeutic target or signaling proteins that are involved in resistance to targeted therapy [182]. The efficacy of kinase targeted therapy in patients with melanoma is influenced by *B-Raf proto-oncogene, serine/threonine kinase* (*BRAF*) and *KIT proto-oncogene, receptor tyrosine kinase* (*KIT*) mutations. Comparison of the mutation profile in CTCs and in the resected primary melanoma showed some heterogeneity [183], particularly for the KIT gene. Therefore, mutation screening is critical to identify patients who will benefit from targeted therapy [183]. Several interventional trials are testing the value of taking into account CTC count (cut-off= 5 CTCs/7.5 mL blood) and phenotype for first-line treatment [184]. A small inversion within chromosome 2p results in the fusion of the *anaplastic lymphoma kinase* (*ALK*) gene with *echinoderm microtubule-associated protein-like 4* (*EML4*) and the production of oncogenic *EML4–ALK* fusion transcripts [185]. *EML4–ALK* analysis in CTCs from patients with NSCLC indicated that *EML4–ALK*+ CTCs are associated with resistance to crizotinib (ALK inhibitor), and thus are a promising candidate for monitoring treatment efficacy and for the early detection of drug resistance [186].

In conclusion, CTC enumeration and mutation profiles give information on the prognosis and help to stratify patients in clinics.

### 4.3. Which One Is Better, CTC or ctDNA? 

Circulating tumor DNA (ctDNA) is actively released from living tumor cells into the blood stream and can be used as a surrogate biopsy biomarker. Its quantification and genomic analysis are useful for the management of patients with tumors in the era of precision oncology [187]. Like CTCs, ctDNA can mirror the tumor burden and stage, and even its metastatic potential, and thus is a promising biomarker for diagnosis, prognosis, and prediction of the therapeutic responses [188]. However, unlike CTCs, the prognostic value of ctDNA is questioned because cell-free DNA, of which ctDNA is only a fraction, is increased also during physiological clonal hematopoiesis, and during pregnancy [187,189]. Moreover, CTCs bring more information (e.g., tumor biology and drug sensitivity evaluation) compared with ctDNA because CTCs can be used in different assays and also in ex vivo models [188]. For instance, immunocytochemistry and FISH cannot be performed on plasma samples for the in situ and morphological analysis of ctDNA [188]. 

On the other hand, it has been reported that ctDNA is much more sensitive than CTCs for the detection of *KRAS* mutations in patients with NSCLC [190]. Also, ctDNA-based tests are better for the precise identification of *EGFR* mutations in patients with NSCLC [191,192]. In patients with NSCLC, CTC- and ctDNA-based tests allow the dynamic monitoring of the disease for targeted therapy and prognosis [192]. The analysis of both CTCs and ctDNA in patients with metastatic solid cancers shows that they represent an effective tool for predicting the risk of tumor relapse and patient outcomes (e.g., OS, PFS) [193]. Moreover, serial deep sequencing of plasma ctDNA from patients with non-metastatic colorectal cancer after adjuvant chemotherapy indicate that ctDNA can detect MRD or clinical relapse in the early phases. It also allows the real-time monitoring of therapy effectiveness, and can help in the choice of targeted therapies after disease recurrence [194]. In conclusion, CTCs and ctDNA are complementary tools with their own specific limitations, and their combined analysis could be useful for cancer management [187].

### 4.4. CTC Targeting for Metastasis Therapy

The higher metastatic potential of CTC clusters compared with single CTCs is associated with poor prognosis [40]. CTC clusters could be targeted with agents that exploit the presence of TAMs [31] and neutrophils [75] in such clusters (e.g., immune checkpoint blockade antibodies and targeting therapy). Moreover, TAM-derived IFNs induce BST-2 overexpression that supports metastasis formation [33]. In a mouse model of breast cancer, BST-2 silencing reduces CTC number and lung metastases [33]. Therefore, BST-2 might represent a candidate therapeutic target for metastatic disease. 

CTC cluster survival in the bloodstream and extravasation are supported by platelets (i.e., “platelet cloaking”) [195]. Disrupting CTC-platelet interaction by blocking platelet function using drug-loaded liposomes conjugated to a tumor-homing pentapeptide (CREKA) could inhibit tumor metastasis formation [196]. However, this approach could also impair platelet normal hemostatic function [197]. Alternatively, TRAIL-expressing platelets could be engineered to kill DR-expressing CTCs and reduce metastasis formation [197]. 

In solid primary tumors, autophagy induces the endocytosis of TRAIL receptors, resulting in resistance to TRAIL-based therapy [27]. However, TRAIL-resistant cancer cells become again sensitive to TRAIL-induced apoptosis after they detach from the ECM and face the hemodynamic shear stress [198,199]. A recent study used liposomes functionalized with TRAIL and E-selectin molecules that bind to circulating leukocytes to significantly reduce tumor growth and CTC number in an orthotopic xenograft mouse model of prostate cancer [200].

Besides metastases in distant organs, the leaky-prone neo-vasculature of the primary tumor and tumor-draining lymph nodes promote CTCs’ re-entry in the tumor of origin after circulation (i.e., “tumor self-seeding”) [15]. Tumor-draining lymph nodes are the first site of metastasis in patients with melanoma, carcinoma, and sarcoma, and they are also reached by CTCs, thus creating an immune-repressive environment that favor tumor spreading [201]. The presence of NK cells with different phenotypes in tumor-draining lymph nodes provide the opportunity to decorate liposomes with TRAIL molecules and NK1.1, an antibody against an antigen expressed by NK cells [202]. TRAIL/anti-NK1.1 liposomes binding to NK cells significantly reduced the incidence of lymph node metastases in mice xenografted with SW620 colon adenocarcinoma cells [202]. Moreover, using blocking antibodies against midkine, a cytokine that induces lymphangiogenesis in distant organs, may suppress lymph node metastasis formation in melanoma [48]. 

As CTCs express CD47, an anti-phagocytic receptor [74] (Figure. 3b), CD47 has been targeted with antagonistic mAbs in animal models [203,204] and clinical trials. However, CD47 targeting causes anemia and thrombocytopenia due to the high CD47 expression on red blood cells and platelets [205]. This effect could be avoided by producing anti-CD47 nanobodies that lack the Fc-mediated adverse effector function [204,206]. Also, engineered bacteria containing a synchronized lysis circuit (eSLC) to locally release a CD47 antagonist can be used to specifically deliver the agent at the tumor site, to induce a systemic anti-tumor immune response and lyse tumor cells in response to diverse tumor microenvironment stimuli [207].

CTC cluster dissociation into single cells with Na+/K+ ATPase inhibitors or by cell–cell junction knockdown allows DNA methylation remodeling at critical sites, highlighting the direct connection between cell clustering and methylation status [20]. Na+/K+ ATPase inhibitors are emerging as a new strategy to limit cancer spread, and these data provide the rationale for testing them in clinical studies. For example, the Na+/K+ ATPase inhibitors ouabain and digitoxin are two FDA-approved agents that can also dissociate CTC clusters into single cells and suppress spontaneous metastasis formation in breast cancer xenograft models [20]. However, these drugs display important cardiac toxicity, limiting their use for cancer management. 

Once dissemination has occurred early in tumor progression, DTCs display fewer mutations [208] and fewer neo-antigens compared with the primary tumor and overt metastases [209]. This offers the prospect for adoptive transplantation of TCR-engineered T cells, synthetic chimeric antigen receptor (CAR) T cells, and memory stem cell (TSCM) and central memory (TCM) T-cell subsets that are not dependent on the MHC-restricted immune response [210,211,212]. Moreover, TSCM and TCM can self-renew and differentiate into effector cells that boost their in vivo durability and therapeutic efficacy [211,212,213]. 

The ‘awakening’ of dormant DTCs and the use of anti-proliferative therapies to eradicate them are clinical paradigms [213]. However, this approach is limited by chemotherapy inefficiency, DTC genetical heterogenicity, and the risk of systemic tumor recurrence [214,215]. Also, druggable modulators only target proliferative CTCs, but not dormant DTCs and the MRD that lead to recurrence decades after therapy [6]. To overcome these limitations, drugs to prolong cancer dormancy could be developed [216]. For example, long-term adjuvant tamoxifen treatment in patients with ER-positive breast cancer reduces recurrence rates, possibly by restraining DTC proliferation [217]. 

Finally, targeting the metastatic niches is another anti-tumor therapy approach. The pre-metastatic niche forms before CTC arrival (e.g., via exosomes) and the post-metastatic niche initiates and takes form upon CTC arrival. As each cancer exhibits a proclivity to metastasize in specific organs, the CTC niche type in distant sites should be characterized [218]. 

## 5. Conclusions 

Understanding CTC biological properties, how they migrate (single cells or clusters) and escape the anti-tumor immunity, as well as their pre- and post-metastatic niche will offer opportunities to improve cancer management.

CTC enrichment and detection technologies have improved in the last decades. For routine use, it would be better to develop technologies that simultaneously enrich and detect CTCs by downstream molecular characterization at the single-cell level. Moreover, CTC detection might be enhanced in the vessels and lymphatic network close to the tumor [219].

The implementation of a tumor-specific signature for CTC identification improves personalized medicine through disease monitoring to guide treatment decisions, and even for metastatic cancer therapy. Targeting CTCs in the blood circulation might represent a promising therapeutic strategy; however, CTC biology needs to be fully understood. Moreover, insights into the role of EMT, stemness, clustering, and immune escape in CTCs help to better understand the metastatic cascade and consequently to guide research on future anti-cancer agents. 

## Figures and Tables

**Table 1:** CTC enrichment and detection based on their (a) biological and (b) physical properties.

Enrichment Method	Selection Criteria	Detection Method	Advantages	Disadvantages	Ref.
**(a) Biological-based methods for CTC enrichment**					
*Immunoaffinity-positive enrichment*					
CellSearch^®^	EpCAM	Flow cytometry	● FDA-approved method, ● Clinically relevant automated system, ● Quantitative, ● Easy, ● Highly robust and reproducible	● Do not detect EpCAM-negative CTCs, ● Expensive and subjective image evaluation, ● Cell preservative limits RNA analysis	[16,18,103,135]
CellCollector^®^	EpCAM	Immunocytochemical staining	● Can isolate rare CTCs in early cancer stages, ● Provide more CTCs, ● No need of blood sampling, ● Detect CTCs in vivo	● Cannot isolate EMT-CTCs, ● CTCs cannot be released from the wire	[115,116]
MagWIRE system	EpCAM	qPCR	● Large-scale CTC capture in vivo, ● Very fast processing, ● Completely self-contained, ● Flexible	●Require additional biocompatibility testing, ● Capture EpCAM-positive CTCs only, ● Possible systemic exposure to excess iron	[116,117]
rVAR2-based CTC isolation Ligand-receptor affinity	Oncofetal chondroitin sulfate (ofCS)	Flowcytometry/ddPCR/Western blotting/Four-color immunofluorescence staining	● Not dependent on the expression of a single marker, ● Low contamination of PBMCs, ● CTC enumeration correlates with disease stage	● Need to be followed by CTC detection methods, ● Need redesign for each tumor type, ● Not commercially available	[137]
***Immunoaffinity-negative enrichment***					
EasySep™	CD45 depletion	Flow cytometry	● Simple, ● Easy-to-use batch separation, ● Do not bias the sample according to selection markers	● False positive results due to CD45^–^ endothelial cells, ● Do not achieve the same high purity levels	[138]
Quadrupole Magnetic Separator (QMS)	CD45 depletion	IF staining	● High-throughput magnetic cell sorter, ● Continuous flow	● RBC lysis required	[139]
**(b) Physical methods for CTC enrichment**					
***Size and deformability***					
ISET technology	Size/ Deformability	IF staining	● Easy and fast processing time, ● Sensitivity threshold of 1 CTC/mL of blood, ● Label-independent isolation, ● Isolation of intact CTCs, ● Isolation of EpCAM-negative CTCs, ● CTCs can be further analyzed by multiplexed imaging and genetic analysis	● Low specificity, ● Retention only of CTCs larger than the leukocyte range, ● Bigger leukocytes may be captured, ● Blood cells need to be fixed, ● False-positive results, ● Low recovery and purity, ● Need the pathologists’ expertise for CTC detection	[3,16,18,140]
Spiral- Slits Chip	Size and deformability	RT-PCR	● Avoid clogging, ● Continuous separation, ● Minimal contamination, ● Detection with optical spectroscopy, ● Fast processing	● False-positive results, ● Need optimizing the structure geometry, ● Low sensitivity	[141]
Cluster-Chip	Strength of cell-cell junctions	RNA sequencing	● Label-free isolation ● Potential study of tumor-immune system interactions ● Chemistry-free approach	● Lack of biological characterization and clinical significance, ● Not commercially available, ● Shear stress is needed to release the majority of clusters from micropillars	[31]
Nanotube-CTC-chip	Preferential adherence or phenotype	IF staining	● Antigen/Size-independent CTC capture, ● Better capture sensitivity from droplets, ● No cell loss, ● Surface architecture lends itself to easier CTC downstream analysis, ● CTC isolation with high purity and 100% sensitivity, ● Can capture CTCs with different phenotypes	● Need development for surface architecture, ● Not commercially available, ● Cannot isolate EMT-CTCs, ● Long set-up times	[141]
***Dielectric properties***					
DEPArray™	Electrical signature		● DEP cages allow the recovery and manipulation of viable single cells	● Requires pre-enrichment	[142]
ApoStream^®^	Conductivity Morphology and Membrane surface area	IF staining	● Label-independent isolation, ● Continuous flow, ● Captures viable cells	● Cells need to be pre-purified because whole blood has high electrical conductivity	[18,143,144]
***Density***					
OncoQuick^®^	Density and filtration	-	● Porous membrane prevent mixing, ● Simple, ● Inexpensive	● Relative low yield and enrichment	[120]
Ficoll-Paque^®^	Density	RT-PCR	● Inexpensive, ● Easy-to-use	● Significant CTC loss	[145]
***Inertial Focusing***					
Labyrinth device	Size	IF staining	Can capture viable cells, label free	Prior RBC depletion required	[130]
Multi-flow microfluidic device	Size and inertial forces	IF staining	Predictable and tunable cutoff size, isolation of CTC clusters, one-step isolation	Relative low flow rate,	[128]
Micro-ellipse filters	Size, deformability and inertial forces	IF staining	Robust, large sample processing, on-chip culture	RBC lysis required	[129]
ClearCell^®^ FX	Size and inertial forces	Flow cytometry	● Can captures viable cells, ● Easy to manufacture, ● Can process a 7.5 mL sample in 8 min, ● Exerts minimal stress on captured cells	● RBC lysis required	[131]
Vortex	Size	IF staining	● No RBC lysis required, ● Can capture viable cells, ● Easy to manufacture, ● Detect clusters	● Low sensitivity and reproducibility	[132]
***Photoacoustic flow cytometry***					
Ultrasound and a pulsed near-infrared laser	-	Flow cytometry	● Can count CTCs in blood vessels up to 3 mm deep, ● Label-free	● Only used for CTC count, ● No molecular analysis	[146]
**(c) Combined methods**					
GEDI chip	Size and immunoaffinity	cDNA sequencing and immunostaining	● Relative high yield and enrichment	● Requires surface chemistry modifications, ● Requires high-resolution imaging method	[147]
CTC-iChip (Microfluidic immunomagnetic-based)	Size inertial focusing Negative enrichment	Mass cytometry	● Allows the sequential separation of different blood components through micropillar array, ● Hydrodynamic size-based sorting/magnetophoresis, ● Simplicity, ● Can sort rare CTCs	● Samples not suitable for DNA sequencing, ● Expensive, ● Long set-up times, ● Difficult to implement in clinical settings, ● Multiple manually interconnected chips	[148]
RosetteSep system	Negative enrichment EPISPOT (protein secretion)	IF staining	● Captures and detects viable CTCs at the single-cell level, ● Does not need whole-genome or transcriptome amplification, ● Limited number of markers	● Proteins must be actively secreted ● Unbiased enrichment independent of CTC/ DTC phenotype	[140,142,149]
RosetteSep system EPIDROP	Secreted proteins Density centrifugation Immunoaffinity	IF staining	● Simultaneous proteomic and secretomic analysis of single viable CTCs, ● Can test different drugs in a single patient, ● More reliable and sensitive than EPISPOT, ● Automatic detection of positive events using the appropriate software	● Prototype development still in progress	[6]

**Table 2:** Examples of clinical trials testing CTC-based enrichment methods.

Device	Ref.	Enrichment/Detection Method	Condition	Status	Primary Endpoints	Trail
GILUPI CellCollector^®^	[116]	Immunoaffinity (anti-EpCAM Ab)	Locally advanced breast cancer	Completed	PFS, OS	NCT03732339
EMT-marker based ferrofluid device	[150]	N-cadherin or O-cadherin based analysis	Metastatic breast and prostate cancers	Completed	Clinical stage, Screening	NCT02025413
ISET^®^ technology	[151]	Immunocytochemistry (PD-L1 expression analysis) FISH analysis of *ALK*	Lung cancer Lung Neoplasms	Recruiting Active	Not provided Validation of *ALK* analysis	NCT02827344 NCT02372448
Culture system	[152]	Affinity-based WBC deletion	Early detection of cancer	Recruiting	Early diagnosis and screening	NCT03843450
Flexible Micro Spring Array (FMSA)	[153]	Filtration (or size-exclusion of viable CTCs)	Stage IV colorectal cancer	Completed	PFS, OS, response to therapy	NCT01722903
Ficoll enrichment	[145]	Density/ PCR	Pancreatic ductal adenocarcinoma	Completed	PFS, OS, response to therapy	NCT02150746
CellSearch^®^	[154]	Immunoaffinity (anti-EpCAM Ab)	Prostate cancer Metastatic breast cancer	Recruiting Completed	EMT markers, PFS and OS CTC-Endocrine Therapy Index	NCT04021394 NCT01701050
CTC-Chip	[102]	Size or Immunoaffinity	Prostate cancer (Prostatectomy)	Recruiting	Examine chromosome translocation	NCT01961713
Parsortix™	[155]	Cellular size and deformability	Ovarian neoplasms	Completed	Estimate risk of cancer	NCT02785731
IsoPic™ microfluidic system	[156]	Flow rate, surface interactions, plasticity, and elasticity	Unknown primary cancer	Recruiting	Prediction of molecularly targeted therapies	NCT04025970
